# Evaluation of the Diagnostic Utility of Selected Serum Adipokines and Cytokines in Subjects with MASLD—A Pilot Study

**DOI:** 10.3390/nu16091381

**Published:** 2024-05-02

**Authors:** Beata Zyśk, Lucyna Ostrowska, Joanna Smarkusz-Zarzecka, Karolina Orywal, Barbara Mroczko, Urszula Cwalina

**Affiliations:** 1 Department of Dietetics and Clinical Nutrition, Medical University of Bialystok, Mieszka I 4B Street, 15-054 Bialystok, Poland; lucyna.ostrowska@umb.edu.pl (L.O.);; 2 Department of Biochemical Diagnostics, Medical University of Bialystok, Waszyngtona 15A Street, 15-269 Bialystok, Polandmroczko@umb.edu.pl (B.M.); 3 Department of Biostatistics and Medical Informatics, Medical University of Bialystok, Szpitalna 37 Street, 15-295 Bialystok, Poland

**Keywords:** hepatic steatosis, NAFLD, MASLD, adipose tissue, visceral adipose tissue, VAT, adipokines, cytokines, Il-6, Il-1β

## Abstract

Excess adipose tissue, particularly of the visceral type, triggering chronic low-grade inflammation and altering its secretory profile, is a contributing factor to the initiation and progression of metabolic dysfunction-associated steatotic liver disease (MASLD). This study aimed to compare the levels of selected adipokines and cytokines in individuals with normal weight and obesity, assessing their potential for diagnosing MASLD and establishing a cutoff point for body fat content associated with hepatic steatosis development. The research involved 99 participants categorized by body mass index and MASLD presence, undergoing body composition analysis, liver elastography, biochemical tests, and evaluation of adipokines and cytokines in serum. The results indicated elevated IL-6 (interleukin 6) serum levels in individuals with obesity with MASLD compared to the normal-weight group without MASLD. The multivariate regression analysis demonstrated a connection between hepatic steatosis and total adipose tissue content, VAT (visceral adipose tissue), VAT/SAT (subcutaneous adipose tissue) ratio, HOMA-IR (homeostasis model assessment of insulin resistance), IL-6, Il-1β (interleukin 1β), and MMP-2 (matrix metalloproteinase 2). Among the adipokines and cytokines examined in this study, interleukin 6 was the strongest predictor of MASLD regardless of gender. In addition, an association between the development of hepatic steatosis and higher serum IL-1β levels and higher adipose tissue was observed in women. However, further studies on a larger group of patients are needed to consider the use of these cytokines as markers of MASLD. The HOMA-IR index demonstrated potential diagnostic utility in identifying hepatic steatosis.

## 1. Introduction

Given that the primary cause of hepatic steatosis, aside from chronic excessive alcohol intake, is systemic metabolic dysfunction stemming from peripheral and hepatic insulin resistance, a proposition emerged in 2020 among global experts to alter the current nomenclature from “non-alcoholic fatty liver disease (NAFLD)” to “metabolic dysfunction-associated fatty liver disease (MAFLD)” [1]. In 2023, there was a renewed proposal to modify the terminology to “metabolic dysfunction-associated steatotic liver disease (MASLD)”. This revised term considers the potential coexistence of these crucial etiological factors—metabolic syndrome and alcohol abuse—in the onset of liver steatosis, as well as the prospect of its development in individuals without obesity [2].

The global prevalence of metabolic dysfunction-associated steatotic liver disease (MASLD) has risen by approximately 50% in the general population over the past 30 years, now impacting up to one-third of the global population [3]. The prevalence is notably higher in patients with obesity, reaching 75.3% [4], owing to the close connection between MASLD pathogenesis and metabolic disorders arising from prolonged positive energy balance, leading to excessive visceral adipose tissue (VAT) and its disorders [5]. Nevertheless, MASLD can also develop in individuals of normal weight or overweight, constituting approximately 18.3% of the adult population [6]. It is hypothesized that these individuals may exhibit increased VAT and a heightened risk of developing metabolic disorders, and relying solely on body mass index (BMI) may not be a reliable marker for diagnosing MASLD [7].

Visceral adipose tissue exhibits significantly higher metabolic activity compared to subcutaneous adipose tissue (SAT). A mere 1% rise in its content results in a substantial 40% increase in hepatic lipid accumulation [8]. Adipose tissue plays a pivotal role in the onset and the progression of MASLD, owing to its capability to release numerous biologically active substances. The surplus adipose tissue, particularly visceral adipose tissue, creates a predisposition for chronic low-grade inflammation, accompanied by alterations in its secretory profile. This involves heightened secretion of leptin, tumor necrosis factor α (TNF-α), and interleukin 6 (IL-6), as well as a reduction in adiponectin secretion [9].

The relationship between leptin and adiponectin and MASLD has been extensively described in the previous literature. However, further research on adiponectin is important, as this adipokine is a promising diagnostic and therapeutic target [5,9]. There are still many adipokines and cytokines whose involvement in the pathogenesis of MASLD and MASH is still unclear, including resistin, TNF-α, IL-6, IL-1β (interleukin 1β), IL-23 (interleukin 23), and extracellular matrix metalloproteinases (more specifically, gelatinase). The assessment of concentrations of specific adipokines and cytokines holds promise as a potential component in the diagnosis of hepatic steatosis. Currently, a very reliable and sensitive method of assessing the degree of steatosis is the transient elastography method [1]. An analysis of the correlation of the degree of hepatic steatosis, as assessed by this method, with serum concentrations of adipokines and cytokines will be helpful in finding potential markers of MASLD. Blood samples for determining these adipokines or cytokines could conveniently be collected alongside other routine blood biochemical tests.

The purpose of this research was to assess and compare the serum levels of specific adipokines and cytokines in individuals with normal weight and obesity, exploring their potential utility in diagnosing MASLD. Additionally, the study sought to determine a cutoff value for body fat content that may contribute to the onset of hepatic steatosis.

## 2. Materials and Methods

**Figure 1:**
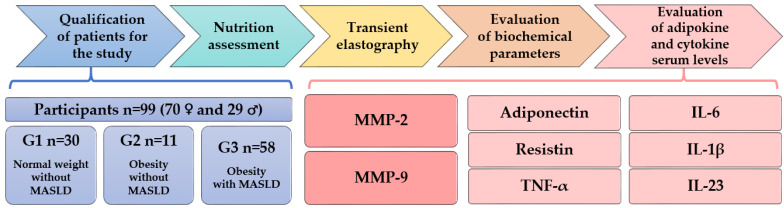
Stages of the observational study.

An observational study was carried out in July and August 2020, with approval obtained from the Bioethics Committee of the Medical University of Bialystok (Approval Nos. RI-002/647/2019, APK.002.468.2020, and APK.002.39.2021). Detailed information about the study was provided to the participants, who were informed of the possibility of withdrawing from the study at any point. Written consent was obtained from all participants. The sequential steps of the study are depicted in Figure 1.

### 2.1. Criteria for Qualifying Patients for the Study

Subjects in the age range of 20–55 years, women and men with normal body weight (BMI = 18.5–24.9 kg/m^2^), as well as primary obesity of the first and second degree (BMI = 30.0–39.9 kg/m^2^), were eligible to participate in the study by random selection. Exclusion criteria for the study included the presence of viral hepatitis (all types), hepatic cholestasis, and daily alcohol consumption >30 g in men and >20 g in women, as well as the presence of type 1 and 2 diabetes mellitus, acute coronary artery disease, eating disorders, endocrine disorders; the use of hormonal contraception or hormone replacement therapy; a history of steroid therapy and antiretroviral therapy; the presence of a pacemaker; and pregnancy and lactation. For participants with obesity, additional exclusion criteria were secondary obesity and pharmacological or surgical treatment of obesity.

Participants were selected based on their medical history, resulting in the inclusion of 107 subjects. They underwent a nutrition assessment and were categorized according to BMI. Thus, the participants were divided into a group of normal weight (*n* = 38) and a group with obesity (*n* = 69). Subsequently, all participants underwent liver elastography to evaluate the presence of hepatic steatosis and fibrosis. Each participant’s blood pressure was measured using an electric blood pressure monitor and selected biochemical parameters were assessed. The next step was to assess the presence of MASLD among all participants based on the latest diagnostic criteria [2]:

Hepatic steatosis identified by imaging and at least 1 out of 5 of the following:

🗸 BMI ≥25 kg/m² or waist circumference >94 cm (men) and >80 cm (women);
🗸 Fasting serum glucose ≥5.6 mmol/L [100 mg/dL] or 2 h post-load glucose levels ≥7.8 mmol/L [≥140 mg/dL];
🗸 Blood pressure ≥130/85 mmHg or specific antihypertensive drug treatment;
🗸 Plasma triglycerides ≥1.70 mmol/L (150 mg/dL) or lipid lowering treatment;
🗸 Plasma HDL-cholesterol ≤1.0 mmol/L (40 mg/dL) (men) and ≤1.3 mmol/L (50 mg/dL) (women) OR lipid lowering treatment.

From the control group (with normal body weight), 8 subjects meeting MASLD criteria were eliminated. The explicit grouping was structured as follows:

▪ Group G1 comprised individuals with normal weight without MASLD (*n* = 30).
▪ Group G2 included those with obesity without MASLD (*n* = 11).
▪ Group G3 encompassed those with obesity and MASLD (with or without hepatic fibrosis) (*n* = 58).

### 2.2. Nutrition Assessment

All subjects taking part in the study had their body weight (to the nearest 0.01 kg) and body height (to the nearest 0.5 cm) measured using a WPT 100/200 OW (RADWAG, Radom, Poland) scale with a height gauge. In addition, the body mass index was calculated from the following formula:

$$BMI=\frac{weight\left(kg\right)}{height{\left(m\right)}^{2}}$$

Study participants underwent a body composition analysis through electrical bioimpedance, and their cross-sectional fat area in the abdominal region was assessed using a BioScan 920-2 device (Maltron, Rayleigh, Essex, UK). The assessments were conducted in the morning, and participants took part in the study after fasting and refraining from vigorous physical activity the day before.

First, the study assessed total body fat (kg, %). A body fat percentage exceeding 35% in women and 25% in men indicates the presence of obesity. The body composition analysis was conducted with participants in the supine position, and electrodes were positioned on the right upper and lower limbs. Subsequently, the abdominal cross-sectional fat area, including visceral (cm^2^, %) and subcutaneous (cm^2^, %), was determined. Values exceeding 120 cm² for VAT and over 225 cm² for SAT are considered elevated [10]. The quantitative analysis of abdominal adipose tissue was performed in the standing position, with electrodes aligned horizontally at the umbilical level. The ratio of visceral to subcutaneous fat (VAT/SAT) was also calculated, with values exceeding 0.9 indicating an increased risk of developing metabolic diseases [10]. The results were processed using Maltron BioScan 920 v1.1 software.

### 2.3. Transient Elastography Measurement of Liver Stiffness and Steatosis

Liver elastography was conducted using the FibroScan 530 Compact device (Echosens, Paris, France). Employing the Vibration-Controlled Transient Elastography (VCTE) method, this device allows for the measurement of liver stiffness. Additionally, it offers the option of assessing the controlled attenuation parameter (CAP) to measure liver steatosis.

Trained operators conducted a series of 10 valid measurements on each participant to ensure the accuracy and reproducibility of the results. Depending on individual anatomical conditions, M- and XL-sized heads were used. The test was performed either in a fasting state or a minimum of 6 h after a meal, with participants in a supine position and the right arm extended and positioned behind the head. An adequate amount of gel was applied before measurement, and the head was positioned in the right intercostal region at the level of the thickest layer of liver parenchyma, avoiding measurement near the liver’s lower or upper edge due to the risk of overestimation in the subcapsular space. During the measurement series, the proper positioning of the probe, i.e., perpendicular to the integuments, was ensured [11].

Liver stiffness (E) correlates with the fibrosis of this organ. The value of this parameter is calculated based on the speed of propagation of the transverse wave, spreading several centimeters into the liver. The transverse wave is generated at a controlled frequency (50 Hz), with the characteristic parameter specific for determining liver stiffness [11].

**Figure 2:**
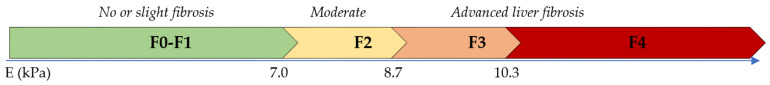
Degrees of liver fibrosis (transient elastography measurement of liver stiffness).

The results obtained were interpreted using a 5-point scale representing the relationship between liver stiffness (in kPa; kilopascal) and the degree of liver fibrosis [12], and the corresponding ranges are illustrated in Figure 2.

Measurement of the CAP^®^ is carried out simultaneously with the measurement of liver stiffness and allows for quantitative assessment of liver steatosis. This parameter is determined by measuring the ultrasound attenuation phenomenon and is expressed in dB/m (decibel/meter). It describes the fading of the ultrasound signal depending on the depth of penetration in the liver.

**Figure 3:**
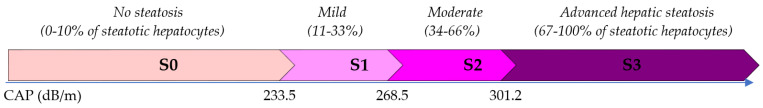
Degrees of liver steatosis (transient elastography measurement of liver steatosis).

The values of the CAP parameter were classified based on the 4-stage liver steatosis scale [13], which is shown in Figure 3.

### 2.4. Evaluation of Biochemical Parameters and Adipokine and Cytokine Serum Levels

#### 2.4.1. Collection of Blood Samples

Blood used in the study was collected in the morning (7:00–10:00 a.m.) from all participants at the same laboratory. Participants were informed in advance about the necessary preparations for the test, which included avoiding heavy, fatty meals and alcohol on the day before the test. Additionally, they were instructed to refrain from increased physical activity immediately before the test. Participants were required to fast before entering the study. Venous blood samples (15 mL) were collected from each patient into a tube with a clot activator (S-Monovette, SARSTEDT, Numbrecht, Germany) and centrifuged to obtain serum samples. Blood serum was utilized for blood biochemical tests, while the remaining quantity of blood serum was frozen at −80 °C for the determination of adipokine and cytokine levels.

#### 2.4.2. Methods for Evaluating Selected Biochemical Parameters

Among the participants, serum concentrations of fasting glucose, glucose after 1 h and 2 h OGTT (oral glucose tolerance test), fasting insulin, insulin after 1 h and 2 h OGTT, total cholesterol, HDLs (high-density lipoproteins), LDLs (low-density lipoproteins), TG (triglycerides), AST (aspartate aminotransferase), ALT (alanine transaminase), GGTP (gamma-glutamyl transferase), ALP (alkaline phosphatase), and CRP (C-reactive protein) were assessed with ALINITY ci-series (Abbott, Wiesbaden, Germany). Concentration of cholesterol was assessed method with cholesterol esterase, HDL by method with accelerator selective detergent, LDL using method with liquid selective detergent, triglyceride with method with glycerol phosphate oxidase, ALT and AST by spectrophotometric method with NADH, GGTP by method with L-gamma-glutamyl-3-carboxy-4-nitroanilide as a substrate, and ALP by method with para-nitrophenyl phosphate. Meanwhile, serum C-reactive protein (CRP) concentrations were determined using turbidimetric method, glucose using method with hexokinase, and insulin using chemiluminescent microparticle immunoassay (CMIA).

#### 2.4.3. Methods for Determining Serum Levels of Adipokines and Cytokines

Serum levels of total adiponectin, resistin, TNF-α, IL-6, IL-1β, IL-23, and total MMP-2 (matrix metalloproteinase 2) and MMP-9 (matrix metalloproteinase 9) were assessed in all subjects participating in the study with the enzyme-linked immunosorbent assay (ELISA) according to the manufactures’ instructions (R&D Systems, Abingdon, UK).

### 2.5. Statistical Analysis of the Results

“Statistica” software (version 13 PL; TIBCO Software Inc., Palo Alto, CA, USA) was employed for the statistical analysis of the results. Due to the small size of one of the groups, it was not possible to assess the normality of the distribution of variables within this group. Therefore, nonparametric methods were used. The Kruskal–Wallis ANOVA test with post hoc analysis was applied to evaluate the significance of quantitative variables, while differences between qualitative variables were assessed using the chi-square test of independence. Multivariate analysis was performed by utilizing multivariate regression modeling the variable that determines the dependent variable (CAP). Regression models were performed for the CAP dependent variable (separate models for women and men), where the independent variables were total adipose tissue (%), VAT (cm^2^), VAT/SAT Ratio, HOMA-IR index, and adipokines and cytokines. For the diagnosis of hepatic steatosis, ROC analysis was performed, and the tangent method was employed to calculate cutoff points for optimal parameter accuracy. The overall effectiveness was expressed by examining the area under the ROC curve with a 95% confidence interval. The statistical significance level was set at *p* < 0.05.

## 3. Results

Table 1 shows the characteristics of the study participants, divided into groups on the basis of body mass index and the criterion for the presence of MASLD.

Over 84% of participants with obesity were diagnosed with MASLD (G3). Individuals in this group were not statistically significantly different from those without MASLD (G2) in terms of gender (*p* = 0.500), age (*p* = 0.185), BMI (*p* = 0.113), total adipose tissue (*p* = 1.000), VAT (*p* = 0.328), SAT (*p* = 0.666), and VAT/SAT ratio (*p* = 0.865). The groups differed significantly in the values of the CAP parameter (*p* < 0.001) and the parameter assessing liver fibrosis, E (*p* = 0.042). In the G3 group, grade I hepatic steatosis was observed in 12 subjects (21%), grade II—in 24 subjects (41%), and grade III—in 22 subjects (38%). Liver fibrosis in this group was observed in 12 people, i.e., 21% (grade I fibrosis—in 4 people (7%); grade II fibrosis—in 6 people (10%); grade III fibrosis—in 1 person (2%); and grade IV fibrosis—also in 1 person (2%)).

Table 2 compares the serum concentrations of selected adipokines and cytokines in the subjects.

Statistically significant differences were observed in interleukin-6 concentrations when comparing G1 vs. G2 (*p* = 0.025) and G1 vs. G3 (*p* < 0.001) groups. However, no statistically significant differences were found in interleukin-6 concentrations between subjects with obesity (G2) and those with obesity and MASLD (G3). There were no statistically significant differences in the concentrations of other evaluated adipokines, cytokines, and gelatinases among all study groups.

Selected blood biochemical parameters were then evaluated and are shown in Table 3.

The comparison of blood biochemical parameters and insulin resistance indices between participants with obesity (G2 vs. G3) did not yield statistically significant differences. However, in the G1 vs. G3 group, statistically significant differences were observed across most studied parameters (in addition to AST, ALP and total cholesterol levels). Elevated fasting insulin levels were found to be a statistically significant differentiator between G2 and G1 patients (*p* = 0.039). Furthermore, these groups exhibited statistically significant differences in the HOMA-IR index (*p* = 0.045) and QUICKI index (*p* = 0.043). The groups also differed in ALT levels (*p* = 0.034).

Table 4 and Table 5 present multiple regression models examining the association between the extent of hepatic steatosis in female and male participants and specific parameters of body composition, as well as serum concentrations of selected adipokines, cytokines, and gelatinases. The tables include full (saturated) models, as well as other models obtained by backward stepwise regression. Empty cells in the table indicate that a particular variable in the model was not used.

It was observed that a higher degree of liver steatosis in women was significantly associated with increased adipose tissue (%), the HOMA-IR index, and a higher concentration of interleukin 1β (pg/mL; picograms/milliliter). It was noted that an increased level of liver steatosis showed a significant correlation with a greater abdominal cross-sectional VAT area (cm^2^) and the HOMA-IR index, along with elevated levels of interleukin 6 (pg/mL) and interleukin 1β (pg/mL). It was found that a heightened level of hepatic steatosis exhibited a notable correlation with an increased VAT/SAT ratio and HOMA-IR index and elevated levels of interleukin 6 (pg/mL) and interleukin 1β (pg/mL), coupled with reduced levels of matrix metalloproteinase 2 (ng/mL—nanograms/milliliter). The 3-factor models confirmed the existence of such relationships.

In the male group, a relationship was noted between a higher degree of liver steatosis and increased total adipose tissue (%). The same relationship was observed for VAT (cm^2^). In addition, based on three-factor models, we found that a higher degree of hepatic steatosis in men was significantly associated with increased adipose tissue (%), and higher interleukin-6 concentration (pg/mL). Moreover, a higher degree of hepatic steatosis was significantly associated with increased VAT area (cm^2^) and higher concentrations of this interleukin (pg/mL).

Subsequently, an ROC analysis was conducted to determine cutoff points for the studied body composition parameters and the occurrence of hepatic steatosis, as presented in Table 6.

ROC analysis revealed significant diagnostic value among women for several parameters: total percent adipose tissue (cutoff point: 40.21%; AUC: 0.94; *p* < 0.001), visceral tissue area in abdominal cross-section (cutoff point: 156 cm^2^; AUC: 0.87, *p* < 0.001), and VAT/SAT ratio (cutoff point, 2.05; AUC, 0.75; *p* < 0.001).

In contrast, for men, the ROC analysis indicated significant diagnostic value only for the visceral fat area (cutoff point, 155 cm^2^; AUC, 0.80; *p* = 0.005).

**Figure 4:**
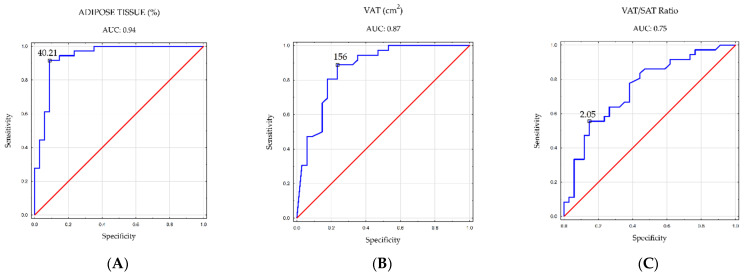
Cutoff points for the incidence of hepatic steatosis in women for (**A**) Total adipose tissue (%); (**B**) VAT (cm^2^); (**C**) VAT/SAT Ratio.

**Figure 5:**
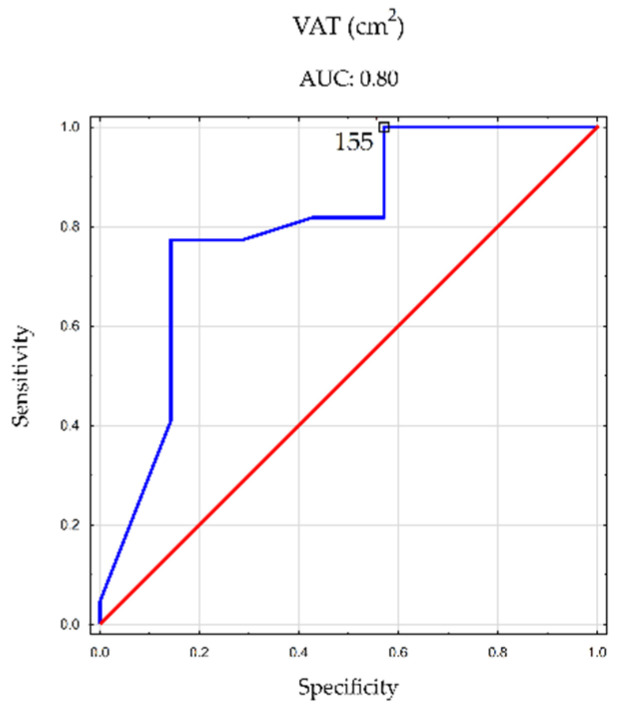
Cutoff points of hepatic steatosis incidence for visceral fat area in abdominal cross-section in men.

Figure 4 and Figure 5 visually depict the cutoff points for the aforementioned body composition parameters, which demonstrated statistical significance in men and women.

ROC analysis was also performed to assess cutoff points for the HOMA-IR index and the incidence of hepatic steatosis. The results are shown in Table 7.

ROC analysis showed a significant diagnostic value for this parameter in both women (cutoff point, 1.81; AUC, 0.79; *p* < 0.001) and men (cutoff point: 1.91; AUC: 0.76; *p* = 0.005).

**Figure 6:**
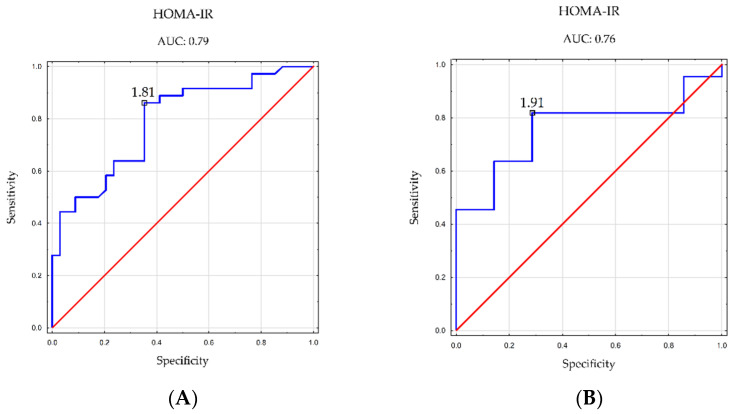
Cutoff points for the incidence of hepatic steatosis for the HOMA-IR index in (**A**) women and (**B**) men.

Figure 6 demonstrates the cutoff points for the HMA-IR index in men and women.

## 4. Discussion

Metabolic dysfunction-associated steatotic liver disease has emerged as a global health concern. The absence of early-stage symptoms poses challenges for diagnosis, leading to incidental identification or detection at advanced stages, such as the progression to steatohepatitis (MASH), liver fibrosis, and potentially cirrhosis. Diagnostic methods for MASLD include computed tomography (CT), magnetic resonance imaging (MRI), ultrasound (US), and elastography (FibroScan). However, their routine use is impractical due to the high costs associated with CT and MRI, despite their high sensitivity. CT also poses the additional drawback of exposing individuals to ionizing radiation. Ultrasound can only diagnose steatosis affecting more than 20% of hepatocytes. Elastography, offering high sensitivity and the capability to detect even minor hepatic steatosis, as well as assess liver fibrosis, is not yet widely available [14].

Currently, there is a lack of effective biochemical tests and markers for diagnosing hepatic steatosis. Our prior research demonstrated that commonly used liver disease diagnostic parameters, such as AST, ALT, GGTP, ALP, and TBIL (total bilirubin) levels, remained within the normal range in most hepatic steatosis patients, making them inadequate for accurate diagnosis [15]. This aligns with findings from other researchers [16,17,18,19]. In recent years, there has been a growing focus on identifying potential markers for metabolic diseases. While previous studies have examined the serum levels of anti-inflammatory and pro-inflammatory adipokines and cytokines in individuals with various conditions characterized by chronic inflammation, such as obesity, type 2 diabetes, and cardiovascular disease [20,21,22], our study stands out as one of the few to investigate the serum adipokines and cytokines profile in patients with MASLD coexisting with obesity.

The literature findings suggest that the presence and progression of MASLD may be indicated by reduced levels of the anti-inflammatory adipokine adiponectin, alongside elevated levels of pro-inflammatory cytokines, including TNF-α, IL-6, and IL-1β. Adiponectin not only diminishes lipid accumulation in adipose tissue and the liver but also enhances liver insulin sensitivity, regulating overall glucose homeostasis. Moreover, it inhibits the proliferation of hepatic stellate cells, pivotal in the development of liver fibrosis [9]. Conversely, the aforementioned pro-inflammatory cytokines activate diverse inflammatory pathways that disrupt insulin signaling [23]. Our investigation also gauged resistin and interleukin 23 levels. Resistin is secreted not only by adipocytes and inflammatory cells but also by hepatic stellate cells. It is believed to primarily impact the liver, with elevated concentrations leading to increased glucose production and the onset of hepatic insulin resistance [24]. Interleukin 23 may play an important role in inflammatory pathways associated with liver damage. According to the results of a recently published study by Yang et al., this cytokine may be a potential marker in the development of fibrosis and cirrhosis [25]. Additionally, metalloproteinases, specifically gelatinases, were selected for evaluation in our study. While matrix metalloproteinases are thought to play a crucial role in liver disease pathogenesis, the precise mechanism remains unclear, with limited studies on the subject. Matrix metalloproteinase 2 (gelatinase A) may contribute significantly to liver fibrosis development, as it is highly expressed in myofibroblasts and is believed to have a profibrogenic role. Matrix metalloproteinase 9 (gelatinase B) is expressed in leukocytes during liver ischemia–reperfusion injury [26].

The results of our study were compared to those of studies other authors on NAFLD (non-alcoholic fatty liver disease), as no such studies on MASLD have yet been established. In addition, 99% of patients with NAFLD meet MASLD criteria [27]. Our study revealed a significant increase in serum IL-6 levels among patients with both obesity and MASLD compared to the normal-weight group without MASLD. Moreover, the assessment of IL-6 levels was found to significantly differentiate between patients with obesity without MASLD and normal-weight individuals. However, serum IL-6 levels did not differ between groups with obesity, both with and without MASLD, indicating that they are not exclusive markers of hepatic steatosis. Baltieri et al.’s study, evaluating IL-6 concentrations in women with obesity post-bariatric surgery with and without hepatic steatosis, found no significant differences between the groups [28]. Duan et al.’s systematic review, encompassing 51 studies, revealed a significant association between increased IL-6 concentrations and an elevated risk of developing NAFLD. However, a careful analysis, considering the clinical subtypes of the disease, indicated that IL-6 concentrations were not significantly linked to steatohepatitis and liver fibrosis [23]. Conversely, Hadinia et al.’s study observed significantly higher levels of IL-6 in patients with NASH compared to a group with NAFL and the group of healthy subjects [29]. Similar results were reported by Fontes-Cal et al. in their study [30]. In our study, there were no significant differences in resistin levels between all groups. Bekaert et al.’s systematic review, encompassing 12 studies on resistin, indicated a significant association between elevated resistin levels and the occurrence or progression of NAFLD in half of the studies [24]. The results from Hegazy et al.’s study demonstrated significantly higher resistin levels in individuals with NAFLD compared to the control group [31]. The variability in findings may imply that increased resistin levels are correlated more significantly with heightened tissue resistance to insulin, thereby elevating the likelihood of hepatic steatosis.

Obesity plays a pivotal role in the initiation and progression of MASLD, with a global systematic review indicating a prevalence of 75.3% among people with obesity [2]. Our study further substantiates this, revealing MASLD in nearly 84% of participants with obesity. Through a tangent ROC analysis, we identified cutoff points for specific body composition components associated with an elevated risk of hepatic steatosis. In women, this threshold was established at 40.21% total body fat, while in men, it was 26.85% (though the diagnostic value was not statistically significant for men). In contrast, Ariya et al. determined cutoffs for total percent body fat at 32.23% in women and 26.73% in men, utilizing dynamic elastography for body composition and dual-energy X-ray absorptiometry scanning for hepatic steatosis assessment [32]. Our study also determined cutoff points for the visceral fat area in the abdominal cross-section, with values of 156 cm^2^ in women and 155 cm^2^ in men. Comparable studies by Yu et al. and Lee et al. in the Asian population, utilizing computed tomography for both visceral adipose tissue and hepatic steatosis assessments, reported different cutoffs. Yu et al. identified cutoffs as 115.55 cm^2^ in women and 178.35 cm^2^ in men [33], while Lee et al. established values of 68 cm^2^ in women and 100.6 cm^2^ in men [34]. The ROC analysis in our study indicated that the VAT/SAT ratio’s cutoff points signaling an increased risk of steatosis were 2.05 in women and 2.04 in men (though diagnostically statistically insignificant for men).

The diminished capacity of adipocytes to store surplus energy in the course of obesity, stemming from a positive energy balance and adipose tissue expansion, results in adipose tissue dysfunction and insulin-resistance development. Accelerated lipolysis in adipose tissue leads to the formation of excess free fatty acids, while the liver accumulates surplus lipids, predominantly triglycerides. Concurrently, heightened lipogenesis in the liver exacerbates fat accumulation. These processes can contribute to the onset of hepatic insulin resistance and hepatic steatosis [35]. Our ROC analysis identified the HOMA-IR index (with a cutoff point of 1.81 in women and 1.91 in men) as a potential diagnostic parameter for hepatic steatosis. Motamed et al., in their study, proposed cutoff points of 1.95 in women and 1.79 in men for the HOMA-IR index in NAFLD diagnosis [36]. Similarly, Isokuortti et al., based on a population-based study, determined 1.9 as the optimal cutoff point for this index, irrespective of gender. Notably, their linear regression analysis indicated that a HOMA-IR value of 2 still corresponded to normal liver fat [37].

Considering the strong correlation observed in our study between various factors, such as obesity, insulin resistance, chronic inflammation, and hepatic steatosis, we conducted multivariate regression analysis to explore their relationship with the CAP-dependent variable. The combined results of the regression models revealed that, in women, a higher degree of hepatic steatosis was significantly associated with an elevated HOMA-IR index, increased percentage of total adipose tissue, greater area of visceral fat in abdominal cross-section (cm^2^), and higher VAT/SAT ratio, as well as higher concentrations of IL-6 and IL-1β and lower concentrations of MMP-2. The results of the regression models in the male group showed that a higher degree of hepatic steatosis was significantly associated with an increased percent body fat, greater area of visceral fat in abdominal cross-section (cm^2^), and higher serum IL-6 levels. Previous research has demonstrated a positive correlation between IL-6 levels and systemic insulin resistance, IL-6 expression in the liver, liver inflammation, and the degree of fibrosis in patients with NAFLD [38,39,40]. A systematic review by Duan et al. indicated a connection between serum interleukin 1β levels and the development of NAFLD, progression to NASH, and the emergence of liver fibrosis [23]. Yilmaz et al. found significantly higher levels of MMP-2 in patients with NAFLD compared to healthy subjects, but these levels did not differentiate between simple steatosis and steatohepatitis [41], consistent with Ando et al.’s study, which also assessed MMP-2 as a potential marker for liver fibrosis progression [42]. Munsterman et al.’s results further supported the association between increased MMP-2 levels and the development of liver fibrosis [43].

While our study has provided valuable insights, it is important to acknowledge its limitations. Notably, the sample size, particularly in the male group, is relatively small. In addition, the number of participants in the groups is unequal to each other. However, this way of classifying participants within the groups was dictated by the purpose of the study. Additionally, our study exclusively utilized the electrical bioimpedance method for assessing body composition, whereas DEXA (dual-energy X-ray absorptiometry) is considered the gold standard with higher accuracy. Although the dynamic elastography method employed in our study exhibits high precision and sensitivity for diagnosing steatosis and fibrosis even in the early stages, it does not permit the assessment of steatohepatitis. To conclusively confirm or exclude the clinical potential of adipokines and cytokines and their utility in diagnosing MASLD, larger-scale studies with more balanced groups, including individuals with and without hepatic steatosis, steatohepatitis, and hepatic fibrosis at various stages, are necessary. The consideration of diverse populations, such as those with normal weight, obesity, or insulin resistance, is also crucial. Furthermore, extensive population-based studies are required to validate the cutoff points established in our investigation.

## 5. Conclusions

Among the adipokines and cytokines examined in this study, interleukin 6 was the strongest predictor of MASLD regardless of gender. In addition, an association between the development of hepatic steatosis and higher serum IL-1β levels and higher adipose tissue was observed in women. However, further studies on a larger group of patients are needed to consider the use of these cytokines as markers of MASLD. The HOMA-IR index demonstrated potential diagnostic utility in identifying hepatic steatosis. The findings suggest that body composition analysis, especially of visceral fat, along with fasting glucose and insulin assessments, including the calculation of the HOMA-IR index, should be integral to systematic screening.

## Figures and Tables

**Table 1:** Baseline characteristics of study participants.

	G1 (*n* = 30) Normal Weight without MASLD		G2 (*n* = 11) Obesity without MASLD		G3 (*n* = 58) Obesity with MASLD					
Parameter	Median	Q1–Q3	Median	Q1–Q3	Median	Q1–Q3	*p*			
							K-W All Groups	Post Hoc		
								G1–G2	G2–G3	G1–G3
Age (years)	35.50	29.00–43.00	39.00	30.00–46.00	46.00	40.00–52.00	<0.001 *	1.000	0.185	<0.001 *
BMI (kg/m^2^)	23.10	22.20–24.60	31.60	31.00–33.00	34.45	32.60–36.60	<0.001 *	0.003 *	0.113	<0.001 *
Total adipose tissue (%)	27.69	22.19–30.39	38.34	33.52–45.15	42.52	33.83–46.91	<0.001 *	0.001 *	1.000	<0.001 *
VAT (cm^2^)	87.50	65.00–132.00	220.00	148.00–261.00	291.00	199.00–350.00	<0.001 *	0.014 *	0.328	<0.001 *
SAT (cm^2^)	66.50	57.00–92.00	112.00	92.00–145.00	135.50	110.00–151.00	<0.001 *	0.004 *	0.666	<0.001 *
VAT/SAT ratio	1.37	0.94–1.62	1.89	1.42–2.51	2.18	1.56–2.86	<0.001 *	0.161	0.865	<0.001 *
CAP (dB/m)	193.50	178.00–214.00	215.00	197.00–229.00	292.50	276.00–315.00	<0.001 *	1.000	<0.001 *	<0.001 *
E (kPa)	3.85	3.30–5.00	4.10	2.80–4.50	5.50	4.30–6.30	<0.001 *	1.000	0.042 *	<0.001 *
	***n***	**%**	***n***	**%**	***n***	**%**				
Women	26	86.67	8	72.73	36	62.07				
Men	4	13.33	3	27.27	22	37.93				

Values are expressed as the median and interquartile range (Q1–Q3) or percentage of respondents (%); *p* *—statistical significance (*p* < 0.05). Abbreviations: BMI—body mass index; VAT—visceral adipose tissue; SAT—subcutaneous adipose tissue; CAP—controlled attenuation parameter; dB/m—decibel/meter; kPa—kilopascal.

**Table 2:** Comparative analysis of concentrations of selected adipokines and cytokines in the study groups.

	G1 (*n* = 30)		G2 (*n* = 11)		G3 (*n* = 58)					
Parameter	Median	Q1–Q3	Median	Q1–Q3	Median	Q1–Q3	*p*			
							K-W All Groups	Post Hoc		
								G1–G2	G2–G3	G1–G3
Adiponectin (ng/mL)	6728.05	4453.80–11,906.50	7448.80	4717.90–8764.00	6283.30	4255.00–9762.30	0.585	--	--	--
Resistin (ng/mL)	9.85	8.02–12.61	14.08	9.47–17.98	11.37	8.82–15.04	0.086	--	--	--
TNF-α (pg/mL)	3.67	2.30–4.84	3.17	0.52–4.95	3.86	2.38–5.79	0.491	--	--	--
IL-6 (pg/mL)	0.70	0.27–1.24	1.53	1.24–2.08	2.00	1.51–2.87	<0.001 *	0.025 *	0.953	<0.001 *
IL-1β (pg/mL)	0.54	0.14–1.22	0.52	0.09–0.63	0.34	0.01–0.90	0.212	--	--	--
IL-23 (pg/mL)	2.40	0.91–4.29	3.33	0.00–6.59	0.71	0.00–2.47	0.029 *	1.000	0.308	0.052
MMP-2 (ng/mL)	399.83	308.68–530.28	375.64	291.78–628.68	339.40	270.76–436.70	0.087	--	--	--
MMP-9 (ng/mL)	859.10	624.10–1083.80	1037.00	628.40–1630.80	811.55	656.10–1091.90	0.560	--	--	--

Values are expressed as median and interquartile range (Q1–Q3). Statistically significant differences between the medians were detected by the Kruskal–Wallis ANOVA test with post hoc analysis; *p* *—statistical significance (*p* < 0.05). Abbreviations: TNF-α—tumor necrosis factor alpha; IL-6—interleukin 6; IL-1β—interleukin 1β; IL-23—interleukin 23; MMP-2—matrix metalloproteinase 2; MMP-9—matrix metalloproteinase 9; ng/mL—nanograms/milliliter; pg/mL—picograms/milliliter.

**Table 3:** Comparative analysis of selected biochemical parameters between groups.

	G1 (*n* = 30)		G2 (*n* = 11)		G3 (*n* = 58)					
Parameter	Median	Q1–Q3	Median	Q1–Q3	Median	Q1–Q3	*p*			
							K-W All Groups	Post Hoc		
								G1–G2	G2–G3	G1–G3
CRP (mg/L)	1.00	1.00–1.30	1.10	1.00–3.70	2.25	1.40–4.10	<0.001 *	0.774	0.132	<0.001 *
Fasting glucose (mg/dL)	91.00	85.00–94.00	95.00	89.00–104.00	96.00	89.00–106.00	0.032 *	0.822	1.000	0.026 *
Glucose after 1 h (mg/dL)	124.50	85.00–145.00	116.00	93.00–138.00	147.00	123.00–182.00	0.001 *	1.000	0.065	0.001 *
Glucose after 2 h (mg/dL)	97.50	84.00–110.00	98.00	80.00–107.00	106.50	98.00–127.00	0.005 *	1.000	0.050	0.019 *
Fasting insulin (µU/mL)	6.25	4.70–8.10	9.40	7.40–12.40	12.35	8.60–15.50	<0.001 *	0.039 *	0.620	<0.001 *
HOMA-IR	1.41	1.03–1.89	2.28	1.67–3.03	2.84	1.93–4.22	<0.001 *	0.045 *	0.645	<0.001 *
QUICKI	0.36	0.35–0.38	0.34	0.32–0.35	0.33	0.31–0.35	<0.001 *	0.043 *	0.840	<0.001 *
AST (U/L)	17.50	16.00–19.00	20.00	18.00–26.00	20.00	16.00–27.00	0.073	--	--	--
ALT (U/L)	15.00	13.00–19.00	23.00	18.00–30.00	25.00	20.00–34.00	<0.001 *	0.034 *	1.000	<0.001 *
GGTP (U/L)	14.00	11.00–17.00	21.00	15.00–28.00	27.50	20.00–43.00	<0.001 *	0.058	0.425	<0.001 *
ALP (IU/L)	53.50	45.00–63.00	52.00	48.00–59.00	62.00	50.00–72.00	0.034 *	1.000	0.150	0.102
Cholesterol-T (mg/dL)	190.50	179.00–231.00	216.00	182.00–232.00	199.50	181.00–231.00	0.562	--	--	--
HDL-C (mg/dL)	61.50	51.00–76.00	55.00	43.00–72.00	48.50	42.00–56.00	0.001 *	0.493	0.712	<0.001 *
LDL-C (mg/dL)	100.50	82.00–115.00	135.00	97.00–163.00	123.00	107.00–154.00	0.001 *	0.050	1.000	0.001 *
TG (mg/dL)	69.00	52.00–85.00	101.00	81.00–123.00	119.50	82.00–163.00	<0.001 *	0.070	1.000	<0.001 *

Values are expressed as median and interquartile range (Q1–Q3). Statistically significant differences between the medians were detected by the Kruskal–Wallis ANOVA test with post hoc analysis; *p* *—statistical significance (*p* < 0.05). Abbreviations: CRP—C-reactive protein; HOMA-IR—homeostasis model assessment of insulin resistance; QUICKI—quantitative insulin sensitivity check index; AST—aspartate aminotransferase; ALT—alanine transaminase; GGTP—gamma-glutamyl transferase; ALP—alkaline phosphatase; T—total; HDL-C—high-density lipoprotein; LDL-C—low-density lipoprotein; TG—triglycerides.

**Table 4:** Selected multiple regression models for the dependent variable CAP (dB/m) in a group of women.

Variable		Model 1	Model 2	Model 3	Model 4	Model 5	Model 6	Model 7
Total adipose tissue (%)	β	3.779			4.072			
	SE	0.666			0.556			
	*p*	<0.001 *			<0.001 *			
VAT (cm^2^)	β		0.296 *			0.259	0.317	
	SE		0.056			0.056	0.057	
	*p*		<0.001			<0.001 *	<0.001 *	
VAT/SAT Ratio	β			23.494				20.387
	SE			5.287				5.182
	*p*			<0.001 *				<0.001 *
HOMA-IR	β	6.371	5.563	7.172	5.854	4.531	5.804	6.200
	SE	2.380	2.501	2.548	2.323	2.509	2.649	2.507
	*p*	0.010 *	0.030 *	0.007 *	0.014 *	0.075	0.032 *	0.016 *
Adiponectin (ng/mL)	β	0.001	0.001	0.001				
	SE	0.001	0.001	0.001				
	*p*	0.078	0.128	0.184				
Resistin (ng/mL)	β	−0.631	−2.049	−2.111				
	SE	1.103	1.135	1.194				
	*p*	0.569	0.076	0.082				
TNF-α (pg/mL)	β	0.158	0.482	0.616				
	SE	1.788	1.832	1.920				
	*p*	0.930	0.793	0.749				
Il-6 (pg/mL)	β	3.370	12.374	16.354		11.692		15.245
	SE	4.138	3.693	3.829		3.441		3.464
	*p*	0.419	0.001 *	<0.001 *		0.001 *		<0.001 *
IL-1β (pg/mL)	β	17.783	17.861	16.165	15.763		13.296	
	SE	6.404	6.583	6.871	5.931		6.564	
	*p*	0.007 *	0.009 *	0.022 *	0.010 *		0.047 *	
IL-23 (pg/mL)	β	−1.630	−0.905	−0.896				
	SE	1.022	1.058	1.111				
	*p*	0.116	0.396	0.423				
MMP-2 (ng/mL)	β	−0.059	−0.074	−0.093				
	SE	0.041	0.042	0.044				
	*p*	0.153	0.083	0.039 *				
MMP-9 (ng/mL)	β	−0.006	−0.016	−0.013				
	SE	0.014	0.015	0.015				
	*p*	0.678	0.287	0.400				
Adj. R^2^		0.554	0.529	0.483	0.536	0.482	0.427	0.442

*p* *—statistical significance (*p* < 0.05). Abbreviations: VAT—visceral adipose tissue; VAT/SAT Ratio—ratio of visceral to subcutaneous fat; HOMA-IR—homeostasis model assessment of insulin resistance; TNF-alfa—tumor necrosis factor alpha; IL-6—interleukin 6; IL-1β—interleukin 1β; IL-23—interleukin 23; MMP-2—matrix metalloproteinase 2; MMP-9—matrix metalloproteinase 9; dB/m—decibel/meter; ng/mL—nanograms/milliliter; pg/mL—picograms/milliliter.

**Table 5:** Selected multiple regression models for the dependent variable CAP (dB/m) in a group of men.

Variable		Model 8	Model 9	Model 10	Model 11	Model 12
Total adipose tissue (%)	β	3.160			3.496	
	SE	1.068			0.886	
	*p*	0.008 *			0.001 *	
VAT (cm^2^)	β		0.303			0.317
	SE		0.114			0.077
	*p*		0.016 *			<0.001 *
VAT/SAT Ratio	β			7.995		
	SE			10.498		
	*p*			0.456		
HOMA-IR	β	−1.855	1.955	6.925	−2.022	0.289
	SE	6.771	6.581	7.343	4.064	3.847
	*p*	0.787	0.770	0.358	0.623	0.941
Adiponectin (ng/mL)	β	0.001	0.001	0.003		
	SE	0.003	0.003	0.004		
	*p*	0.801	0.711	0.514		
Resistin (ng/mL)	β	0.069	−1.089	0.367		
	SE	1.686	1.866	2.078		
	*p*	0.968	0.567	0.862		
TNF-α (pg/mL)	β	2.371	−1.796	1.316		
	SE	3.171	3.680	4.176		
	*p*	0.464	0.631	0.756		
Il-6 (pg/mL)	β	16.647	21.978	10.523	19.275	22.752
	SE	10.741	11.932	12.858	7.521	7.500
	*p*	0.139	0.082	0.424	0.017 *	0.006 *
IL-1β (pg/mL)	β	15.213	13.921	21.758		
	SE	20.498	21.392	25.280		
	*p*	0.468	0.523	0.401		
IL-23 (pg/mL)	β	0.969	0.577	1.165		
	SE	0.913	0.976	1.102		
	*p*	0.302	0.562	0.304		
MMP-2 (ng/mL)	β	0.021	−0.028	−0.071		
	SE	0.093	0.093	0.110		
	*p*	0.828	0.765	0.527		
MMP-9 (ng/mL)	β	−0.006	0.011	0.006		
	SE	0.021	0.023	0.028		
	*p*	0.795	0.651	0.825		
Adj. R^2^		0.337	0.291	0.045	0.413	0.432

*p* *—statistical significance (*p* < 0.05). Abbreviations: VAT—visceral adipose tissue; VAT/SAT Ratio—ratio of visceral to subcutaneous fat; HOMA-IR—homeostasis model assessment of insulin resistance; TNF-α—tumor necrosis factor alpha; IL-6—interleukin 6; IL-1β—interleukin 1β; IL-23—interleukin 23; MMP-2—matrix metalloproteinase 2; MMP-9—matrix metalloproteinase 9; dB/m—decibel/meter; ng/mL—nanograms/milliliter; pg/mL—picograms/milliliter.

**Table 6:** ROC analysis for selected body composition parameters in men and women to determine cutoff points for the incidence of hepatic steatosis.

	Women (*n* = 70)	Men (*n* = 29)
Parameter	Total adipose tissue (%)	
AUC (95% Cl)	0.94 (0.88–1.00)	0.77 (0.49–1.00)
*p*-Value AUC	<0.001 *	0.063
Cutoff point	40.21	26.85
Sensitivity	92%	100%
Specificity	91%	71%
	**Women (*n* = 70)**	**Men (*n* = 29)**
**Parameter**	**VAT (cm^2^)**	
AUC (95% Cl)	0.87 (0.78–0.95)	0.80 (0.59–1.00)
*p*-Value AUC	<0.001 *	0.005 *
Cutoff point	156	155
Sensitivity	89%	100%
Specificity	77%	43%
	**Women (*n* = 70)**	**Men (*n* = 29)**
**Parameter**	**VAT/SAT Ratio**	
AUC (95% Cl)	0.75 (0.63–0.86)	0.58 (0.29–0.86)
*p*-Value AUC	<0.001 *	0.591
Cutoff point	2.05	2.04
Sensitivity	56%	82%
Specificity	85%	57%

Abbreviations: AUC—area under curve; Cl—confidence interval; VAT—visceral adipose tissue; VAT/SAT Ratio—ratio of visceral to subcutaneous fat. *p* *—statistical significance (*p* < 0.05).

**Table 7:** ROC analysis for the HOMA-IR index in men and women to determine cutoff points for the incidence of hepatic steatosis.

	Women (*n* = 70)	Men (*n* = 29)
Parameter	HOMA-IR	HOMA-IR
AUC (95% Cl)	0.79 (0.69–0.90)	0.76 (0.58–0.94)
*p*-Value AUC	<0.001 *	0.005 *
Cutoff point	1.81	1.91
Sensitivity	86%	82%
Specificity	65%	71%

Abbreviations: AUC—area under curve; Cl—confidence interval; HOMA-IR—homeostasis model assessment of insulin resistance. *p* *—statistical significance (*p* < 0.05).

## Data Availability

The data presented in this study are available on request from the corresponding author. The data are not publicly available due to privacy and ethical.
